# Overcoming Clinician and Parent Ambivalence: General Practitioners' Support of Children of Parents With Physical or Mental Illness and/or Substance Abuse

**DOI:** 10.3389/fpsyt.2018.00724

**Published:** 2019-01-08

**Authors:** Marit Hafting, Frøydis Gullbrå, Norman Anderssen, Guri Rørtveit, Tone Smith-Sivertsen, Karin van Doesum

**Affiliations:** ^1^Regional Center for Child and Youth Mental Health and Child Welfare, Uni Research Health, Bergen, Norway; ^2^Research Unit for General Practice, Uni Research Health, Bergen, Norway; ^3^Department of Psychosocial Science, University of Bergen, Bergen, Norway; ^4^Research Group for General Practice, Department of Global Public Health and Primary Care, University of Bergen, Bergen, Norway; ^5^Clinical Psychology, Radboud University Nijmegen, Nijmegen, Netherlands; ^6^Impluz, Prevention Team Mental Health, Deventer, Netherlands; ^7^Regional Center of Child and Youth Mental Health and Child Welfare, Artic University of Tromsø, Tromsø, Norway

**Keywords:** children as next of kin, parents with impairments, general practitioners, health prevention, health promotion, qualitative research

## Abstract

Children who are next of kin to parents with physical or mental illness and/or substance abuse need access to mental health support and several cost-effective interventions are available. Because most parents in the target group often consult general practitioners (GPs), GPs may have a crucial role in identifying burdened children and ensuring their follow-up. However, this important topic has received little attention in clinical discussions and research. In response to the knowledge gap, we conducted the research project Burdened Children as Next of Kin and the General Practitioner. Four sub-studies have been completed and published: a sub-study with qualitative analysis of focus group interviews with GPs (paper 1), a qualitative analysis of focus group interviews with adolescents as next of kin (paper 2), and a qualitative analysis of individual interviews with parents with illness and/or substance abuse (paper 3). The results from these sub-studies were incorporated in a survey sent to members of a nationwide GP organization (paper 4). The aim of the present sub-study was to gain further knowledge about conditions for the encounters between GPs and parents with impairments to be supportive for the children as next of kin. The material of the present sub-study derived from the project's four previous sub-studies and comprised a secondary analysis of the four prior sub-studies. We conducted an overarching thematic analysis of these sub-studies' results sections. We searched for statements from the GPs, the adolescents, and the parents on their experiences and evaluations of the needs of the children and their families, and the possible ways of accommodating these needs in general practice. The analysis shows that both GPs and parents were ambivalent about addressing the topic of the patients' children during consultations. This was the case although the GPs were in a good position to identify these vulnerable children, and the parents were worried about their children's situations. Possible strategies for GPs to overcome this ambivalence can be to (1) strengthen their competence in the topic, (2) gradually build trusting relationships with parents, and (3) gradually gain contextual knowledge about the families' situations. GPs can do this by performing ordinary GP tasks and acknowledging the parents' efforts to give their children good daily lives.

## Introduction

Approximately 20–30% of children 0–18 years at some point in time experience a parent with physical or mental illness and/or substance abuse (1–4). While the prevalence of children living with parents with illness and/or substance abuse varies, as the definitions of these parental problems are often not the same, the negative effects that this situation can have on the children are clear. These children are at risk of poor psychosocial outcomes and health problems (5–7). In addition, the children describe difficulties in their daily lives, health problems, school problems, loneliness, and instability in the family situation (8–13).

During the last few decades, children's legal rights have been strengthened (14). In Norway, a law was enacted in 2010 concerning care for children as next of kin to parents with severe physical illness, mental illness, and substance abuse. Health personnel who have parents in treatment are obligated to inform their children (with their parents' informed consent) about their parents' situations and ensure follow-up if necessary (15). The same year, Sweden enacted an identical law (16).

Families affected by illness and substance abuse face many challenges that can be exacerbated by stigmatization, parental prognosis, exposure to violence and trauma, and the impairment's impact on the family economy. Children as next of kin to parents with the abovementioned problems experience varying risk and protective factors, and the impairments have different impacts on their lives and developments. Despite these differences, the children have in common, at least in periods, that their parents often struggle to fulfill their parental tasks and give their children the developmental support that they need (17, 18). They are children at risk but are often described as “invisible” in public life and to support services, and thus are difficult to reach for health promoting and prevention (17, 19). Identifying these children and giving the family necessary support is an important preventive task. Evidence-based interventions are available (7, 20–22), including home visits, individual and group sessions for parents, support groups for children, and family intervention programs. However, there is a need for more high-quality studies on the effectiveness of these programs and how they adapt to different children and family situations (23, 24).

A study from the United Kingdom (UK) reported that 23% of children between 9 and 17 years of age with mothers with depression met the criteria for a DSM IV diagnosis. However, only 33% of the children with a diagnosis had been identified and were in treatment (25). The mothers were mainly recruited from general practices. The authors called this a situation of missed opportunities because the professionals in contact with the mothers could have identified these children and offered them appropriate follow-up. It was our belief that general practitioners (GPs) might be in a good position to change this situation. Most patients with parental responsibilities who suffer from mental illness, severe somatic illness, and/or substance abuse will repeatedly consult a GP, often the same GP, several times over the years (26). Although this topic has been raised in clinical discussions and research (27–29), there is still a knowledge gap concerning how the GPs could support patients' children as next of kin. Therefore, we conducted the research project Burdened Children as Next of Kin and the General Practitioner. The results of four sub-studies have been published (10, 30–32).

In these four studies, we found that the GPs were in a good position to identify the children, but they experienced substantial obstacles to ensure them follow-up care. Parents and adolescents, however, wanted the GPs to address their family situations in the encounters. This had to be enacted within a trusting relationship where parents and adolescents felt that their struggles to manage their challenges were recognized.

This article presents the results from the fifth sub-study of the project. This is a thematic analysis of the four sub-studies' result sections. The aim is to gain further knowledge about how GPs can take on a child-focus and support children as next of kin when their parents with illnesses and/or substance abuse seek the GP for their own health problems.

### Context and Setting

In Norway, where this project took place, general practice provides an open access for people with all types of health-related problems, and the GPs are often the patients' first medical contact within the healthcare system. GPs cooperate with others in the primary healthcare setting, giving the patients coordinated care. They also act as gatekeepers and coordinate entrance into secondary care by referrals. A patient list system is operational, and almost all citizens are enlisted with a personal GP, which facilitates continuity of the doctor-patient relationship. The children are usually enlisted with their mothers' GP. GPs primarily work at their office in which they receive patients for consultations.

### Theoretical Assumptions

The prevailing consultation model among Norwegian GPs is the patient-centered consultation model (33) which encourages the GPs to explore and understand the patient's expectations, background, and feelings. Then, in dialogue with the patient, the GPs combine these insights with the examination-results and his or her medical knowledge. According to this model, the GPs must explore the context, including family matters and the children's situations. However, if the patients for some reason do not want the GPs to address the children's situations, the subject might be omitted even though the GPs ethically and legally have obligations toward the children.

Patient trust is often a precondition for the patient to allow for and engage in difficult conversations about vulnerable themes with a medical professional (34, 35). In a study from Skirbekk et al. on patient-GP consultations (36), trust is conceptualized as the patient's implicit willingness to accept the physician's judgment in matters of concern to the patient. Several studies on general practice concluded that an attitude of recognition from the GP can encourage the patient to share his or her story (37, 38). Here, an attitude of recognition is described as relational, mutual, and based on respect for the person as a subject, as an authority of one's own experiences. This attitude may make it easier for the GP and the patient to tolerate their different viewpoints during the encounter. The concepts of patient-centeredness, patient trust, and patient recognition provide theoretical support for our analysis (39).

As this project's sub-studies were completed, the researchers discussed the implications of the results. GPs mostly meet their patients in a doctor's office. These consultations between the GPs and the parents are the central arena that can allow the GPs to learn about the children's situations and, in collaboration with the parents, ensure follow-up care of the children. Our research suggests that while the GP may be a crucial figure in helping the children of parents with impairments, there are many missed opportunities in practice (31). Therefore, this secondary analysis of our results might deepen the understanding of the possibilities and limitations of these consultations.

## Materials and Methods

There are four sub-studies in our project considered in the present analysis (10, 30–32) (Table 1): a qualitative analysis of focus group interviews with GPs (paper 1), a qualitative analysis of focus group interviews with youth as next of kin (paper 2), a qualitative analysis of individual interviews with parents with illnesses and substance abuse (paper 3), and a survey sent to members of a nationwide GP organization that incorporated the results of the previous sub-studies (paper 4). The current sub-study comprised a secondary analysis of the four prior sub-studies. The results sections of these four sub-studies provide the data analyzed in the present study. The empirical material of this study allows us to combine the perspectives of GPs, youths and parents on how to support burdened children. To reach a deeper understanding of this topic, a thematic analysis (40) is appropriate as it allows for a systematic treatment of the material. This method involves searching the dataset to find repeated patterns of meaning. The analysis is structured and consists of different steps, as described below. During the analysis the researcher continuously compared the codes and themes they developed with the text as a whole to ensure that the results were grounded in the original data set.

**Table 1:** Overview of the four articles in the project Burdened Children as Next of Kin and the General Practitioner.

	**Aim**	**Design/data collection**	**Participants**	**Parental problems**	**Analysis**
Paper 1	Explore GPs thoughts and experiences with handling the special needs of children as next of kin in general practice	Qualitative interview study/4 focus group interviews	27 GPs, 9 women, 38–65 years of age, 6–33 years in GP		Thematic analysis (40)
Paper 2	Explore significant experiences of adolescents as next of kin that the GP should identify and recognize	Qualitative interview study/4 focus group interviews	15 adolescents, 12 women, 16–25 years of age	5 physical illness5 substance abuse5 mental illness	Systematic text condensation (41)
Paper 3	Identify important factors for the GP to bear in mind during encounters with ill and substance-abusing parents to enable the GP to provide appropriate support to the children	Qualitative interview study/Individual semi-structured interviews	12 parents with a total of 28 children, 9 women	4 physical illness2 substance abuse8 mental illness	Systematic text condensation (41)
Paper 4	Investigate the experiences of GPs concerning their involvement with their ill patients' children and their evaluation of the opportunity to help these children	Web-based survey with some open questions	499 GPs 244 men		Numeric data analyzed by cross tables, t-tests, chi-square testing and multiple regression. Text material from the open questions analyzed by thematic analysis (40)

**Figure 1:**
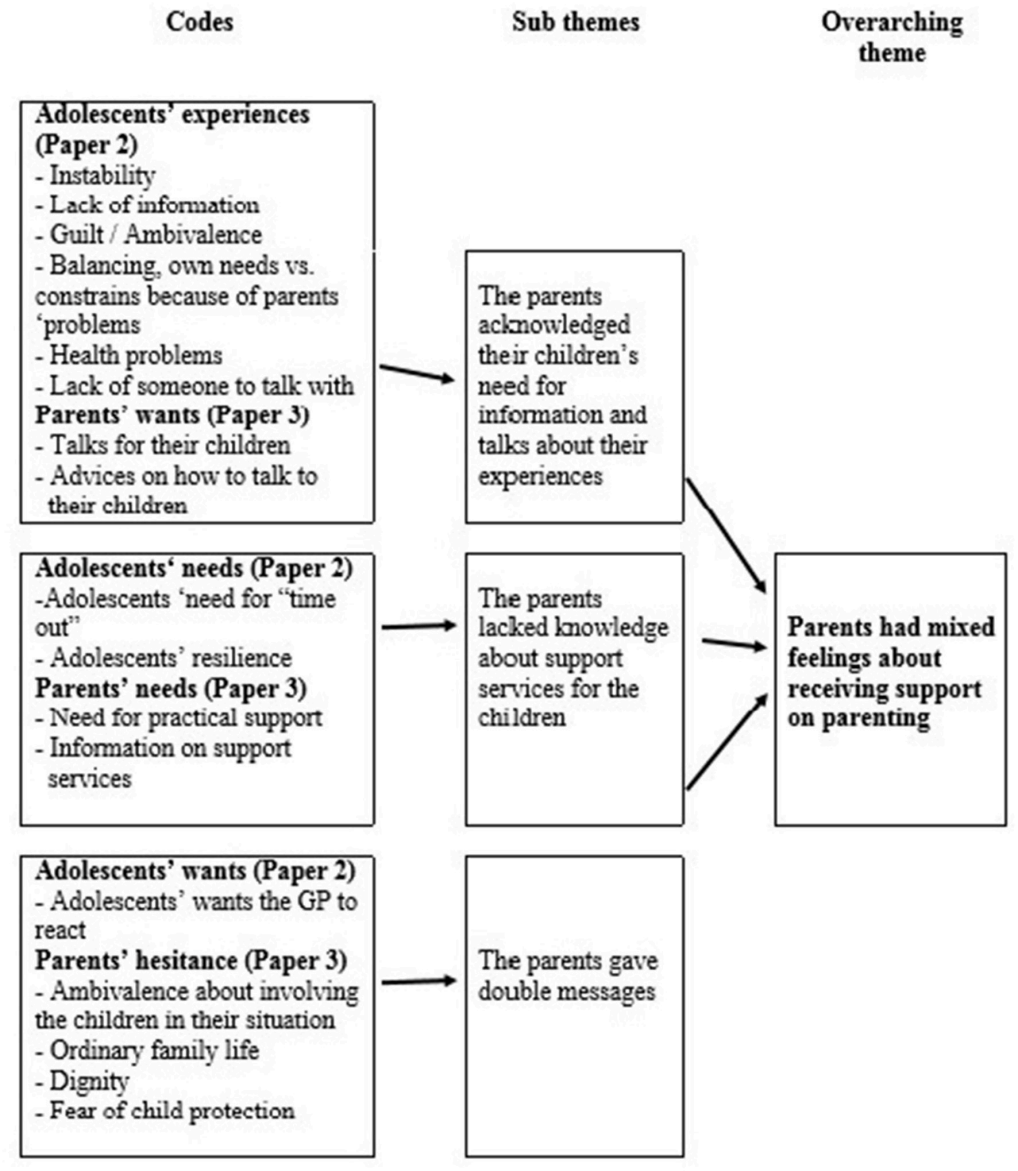
Analysis process from codes over sub-themes to overarching themes. Result section “Patients had mixed feelings about receiving support on parenting” as an example.

After defining the aim, two of the authors (MH) and (FG) read the four articles several times to *familiarize* themselves with the material seen as a whole and recorded their preliminary ideas. Then we searched the results sections specifically for elements that described the informants' experiences and evaluations of the needs of the children and their families, and the possible ways of accommodating these needs in general practice. We marked these elements with *codes*. We found that the text dealt with 37 different codes relevant to our aim. We gathered the textual elements with the corresponding codes. We then determined how the codes from the different sub-studies were interconnected by comparing and integrating them, and then developed preliminary subthemes and overarching themes. Each *subtheme* should cohere together meaningfully, and there should be clear distinctions between them. This process ended with 11 subthemes. We grouped the subthemes under three *overarching themes* that organize the results section of this analysis. The 11 subthemes are used as subheadings (Figure 1). To assure the quality of this analytic process, a third author (KvD) compared the four sub-studies with the list of codes and development of subthemes. At this stage, we searched the text for citations that would best illustrate the subthemes. During this analysis, we evaluated the developed codes, subthemes, and overarching themes against the four sub-studies to ensure that they were based on the empirical material.

## Results

The adolescents described a lack of knowledge about their parents' situations and that they wanted to be offered follow-up on their often-stressful life situations. During the analysis, we found that their needs were acknowledged by the GPs and the parents, but both groups expressed ambivalent feelings about addressing the children's situations in the consultations.

The major overarching themes and sub-themes derived from analysis are (1) The parents had mixed feelings about receiving support on parenting (sub-themes “Parents acknowledged their children's need for information and to talk about their experiences,” “Parents lacked knowledge about support services for their children,” and “Parents gave double messages”), (2) GPs often missed their opportunities to support parents and children (“GPs faced obstacles in the general practice framework,” “GPs feared jeopardizing the doctor-patient relationship,” and “GPs lacked knowledge about talking to the children”), and (3) How can the GP lay the ground for reduced ambivalence of talking about the children? (sub-themes “Recognizing the parents' struggle for an ordinary family life,” “Taking the initiative,” “Awareness of the therapeutic alliance,” “Gaining contextual knowledge and building multidisciplinary networks,” and “Building competence”).

### Overarching Theme 1: The Parents had Mixed Feelings About Receiving Support on Parenting

#### Parents Acknowledged Their Children's Need for Information and to Talk About Their Experiences

During the focus group interviews, the adolescents indicated that they had incomplete knowledge about their parents' conditions and expected outcomes. This caused worries and uncertainty and made their daily lives unpredictable. They described a daily struggle to balance their own needs for an ordinary adolescence—participating in social activities and focusing on school performances—with the boundaries and burdens caused by their parents' problems. For these adolescents, it was important to have someone to talk to about their family situation and who acknowledged their challenges. This individual could be the healthy parent, a teacher, a friend, a family member, a support group, and someone from the health care system, including the GP. A girl with a mother with mental illness, now living in a foster home, said the following (paper 2):

It is so nice to talk to some adults who can tell you that this is NOT how you should live. You should not wash the dishes after a huge dinner that you didn't eat. That is not how life should be for a kid. You should be out playing, because it is sunny outside. That kind of information is incredibly important.

The majority of parents recognized their children's lack of information and emotional burdens. However, giving such information to the children was perceived as difficult. Often, due to the parents' own medical condition, they did not sense their children's worries. For example, consider the statement from one mother with severe chronic back pain (paper 3):

…….because all kids get worried when the mother stays in bed all day, and when they peep into the bedroom, she is laying there crying with pain. Of course, my kids got worried. They were terrified. They thought that I would die. They did not see the difference whether I laid there not being able to move because of back pain, or if I had cancer. For them, there was no difference. I did not manage to sense these worries.

Some parents expressed that their children avoided talking about their parents' problems at home. Often, choosing the best time to inform the children of their problem was difficult. Moreover, the parents were unsure what information was relevant to share. Many parents wanted concrete and individualized advice on how to talk to their children about their situations. For this purpose, they said that a helper close by would be the best person to seek counseling from. Some parents wanted their children to be offered help from professionals.

#### Parents Lacked Knowledge About Support Services for Their Children

The adolescents described constraints in their lives caused by their parents' impairment. It was important for them to relax and take part in social activities such as going out with friends and joining sports activities. They needed “time-outs.” One 16-year-old girl living alone with a father with schizophrenia echoed this sentiment (paper 2):

I just wanted to have time out with them, my friends, where nobody knew about my dad. I found that relaxing. I didn't want to be pitied for living with him, I just wanted to be seen as an ordinary girl.

In addition, parents missed having relieving activities for themselves and their children, help for daily household concerns, and financial support. They often did not have the energy, social network, or competence to search for support services. Parents wanted their GP to ask questions about their family's needs and to help them to find relevant support. A mother with bipolar disorder who lived alone with two children said the following (paper 3):

It is important that the GPs have knowledge about where they can recommend us to get help when it comes to the children. Once the doctor knows that we have children, there should be an alarm ringing telling them: “Okay, now these kids need to be protected.” The doctor should tell the parents: “I have some advice for you, and some helpers you can contact, and here are the phone numbers,” …. a brochure to hand out or stuff—I think that can be very helpful.

#### Parents Gave Double Messages

The youths often wished for more information and to have talks about their life experiences. In particular, they wanted to know more about their parents' conditions, how to understand their sometimes-deviant behaviors, their disease progressions and help to understand their own life situation and the rationale for their struggles. They wanted someone to talk to about their experiences. The parents acknowledged their children's needs to be informed and supported. In addition, they realized that advice on parenting could be beneficial. Despite this, they revealed ambivalent stances on the topic and gave what could be termed a double message; they both wanted and did not want their children's situation to be a topic in the consultations. Some openly stated that they were afraid of being considered bad parents. Likewise, some feared their children would be taken from them if they revealed the problems at home. Some GPs in the survey noticed this resistance and attributed it to the parents' fear of losing custody. The adolescents, however, wanted the GPs to intervene. For instance, one girl blamed the GP for not contacting the child protection services after a consultation she had attended with her mother who was heavily drunk. Related to this, many parents wanted to preserve their dignity and social acceptance. They described their struggle to keep up the appearance of an ordinary family life. Last, the parents were unsure about how much information should be provided to the children, as knowing too much about their parents' problems could cause their children unnecessary concerns. A single mother with a personality disorder living with an 8-year-old son maintained, as did many parents, that keeping up the impression of a normal family was in a child's best interest (paper 3):

I have simply avoided talking about it. I have been afraid about making our situation abnormal, that he might think we are living differently. That he would be ashamed. For this is what is normal for him.

### Overarching Theme 2: GPs Often Missed Their Opportunities to Support Parents and Children

#### GPs Faced Obstacles in the General Practice Framework

Some GPs knew their patients' children from local communities and from collaboration with the health visitors in the geographic area. Others pointed out that they only had a “peephole” into their patients' everyday lives, and the patients regulated the GPs' knowledge of their families. As one GP said: “It is easy to hide from a GP.” Due to the continuity of care, this “peephole” sometimes gradually broadened through knowledge gained over time during ordinary GP tasks.

In particular, the GPs emphasized three factors within the GP framework that hampered their opportunities to address the children's situations during consultations: busy practices with heavy workloads, short consultations, and the registry system where family members could be enrolled with different GPs. Most of the respondents in the survey (paper 4) answered that they felt responsible for their patients' children when they were enrolled on their own list. However, only half of the GPs felt the same responsibility when the child was enrolled with another GP. The following glimpse from an interview between the interviewer (FG) and a GP illustrates this point (paper 1):

FG: “Does the general practitioner already have so many tasks that this becomes difficult to handle during the workday?”

GP: “I think that's a good point, especially in a situation where the rest of the family is not on your list. Then you think there are other people involved who will take care of them. The rest of the family can be people you don't know and whom you have never seen.”

However, these children might have GPs who do not know about their parent's problems. If a child's GP does not routinely ask about his or her parents' condition, this GP may not recognize if the child is at risk. This is often the situation for children of ill or substance-abusing fathers because at birth Norwegian children are automatically enrolled with the same GP as their mother.

#### GPs Feared Jeopardizing the Doctor-Patient Relationship

Relational constraints—such as focusing on the parents during the consultations and thus, forgetting to address the children—caused problems for the GPs. In addition, the GPs were also concerned about the possibility of hurting or losing their vulnerable patients if they brought up the children's situations. Some GPs said that they avoided the topic because they did not want to add guilt and place burdens on parents who were already struggling. One female GP expressed this notion (paper 1):

It is difficult, because then it's as though I am also saying that her problem is her children's problem. Then I am putting the blame on her, and here she has come to get help for herself. I am just placing one more burden on her shoulders, I should think.

Similarly, some GPs avoided the topic because they thought they could offend their patients. These GPs viewed that ending a long-term doctor-patient relationship would be a disadvantage for the patient and the family.

#### GPs Lacked Knowledge About Talking to the Children

The GPs often engaged in discussions about parenting, giving advice to both ill and healthy parents. A female GP told a relevant story about a family with an 8-year-old boy (paper 1):

The mother had asked me for advice on how to inform her son about the father's drug problem. (….) Then, the father died in an overdose. Afterwards I gave advice on how to tell this son about death and why it happened. Naturally, I also visited their home a few times after he died and talked with the little 8-year-old boy. That was not easy!

The GPs who addressed the children's situations were confident in informing and advising their parents. However, GPs felt more uncomfortable about talking directly to the children.

### Overarching Theme 3: How Can the GP Lay the Ground for Reduced Ambivalence of Talking About the Children?

The parents gave a double message on whether to thematize the children's situations in the encounters with the GPs. The GPs said that they often avoided the topic of their patients' children out of fear of placing more burdens on their struggling patients or of losing the therapeutic alliances. However, the data-set contained thoughts and experiences from both the parents and the GPs on how to overcome this mutual ambivalence who might hamper identification of and support to the children.

#### Recognizing the Parents' Struggle for an Ordinary Family Life

Many parents tried to make ordinary daily lives for their children. Overall, they wanted their impairment to have as little negative impact as possible. Parenthood gave them social belonging and self-respect. Crucially, it sent a message to them and those around them that they managed parenting despite their problems. One mother with substance abuse framed it this way (paper 3):

For the last 6 months, a woman from child protection has been coming to my home twice a week to take urine tests. In addition, she does an inspection in our home. I wanted it that way. I wanted these people to come home to me, to let them see that we manage just as well as our neighbors.

For these parents, admitting that they needed support threatened their self-image of being a competent parent who managed daily life in an ordinary family. Therefore, before they could admit their shortcomings and collaborate with the GP, many parents needed recognition for their efforts and love of their children.

#### Taking the Initiative

Despite their expressed ambivalent feelings, parents interviewed in Paper 3 wanted their GPs to address their children's situations. However, the parents did not want to put forward the topic themselves. Rather, they had to be prompted by the GP. The adolescents expressed the same sentiment: They wanted the GP to ask about their family and situation at home during ordinary consultations. The adolescents' expectations of the GP could be negatively formed by their parents' previous medical experiences such as delayed cancer diagnosis or psychiatric diagnoses. The GP might misunderstand this hesitant attitude or ambivalence from parents and children as avoidance or that the topic was not relevant.

#### Awareness of the Therapeutic Alliance

Sometimes, the GPs worried that their rapport with patients would be affected negatively if they addressed the children's situations (see above “GPs feared jeopardizing the alliance”). The parents, however, told stories of how they had previously tolerated direct speech from a trusted helper, someone whose alternative viewpoints and corrections they could accept. The parents often had a trusting relationship with one professional, a person who had provided continuity of care and demonstrated strong personal involvement. One father who had recently lost his wife to cancer expressed this view (paper 3):

Support from the GP, a cancer nurse, or health visitor is really important. To have helpers genuinely interested in helping you and not just doing a job because it is their duty to do so. You tell more to a person you know and trust than to a person you see only once. These helpers have been there during the illness. It started with the GP, the GP has been there all the time, and it is there you go if new troubles come up.

#### Gaining Contextual Knowledge and Building Multidisciplinary Networks

It was important for the parents to have helpers who knew their situation well, including the social and family settings. In addition, the parents appreciated when the GP had adequate knowledge about support services and participated in multidisciplinary meetings. In these meetings, the GP could contribute with valuable information. For the parents, it was important that the different helpers collaborated, as stated by a single mother of two children (paper 3):

My GP is very active participating in collaborative meetings. Then she gets more information about my situation—more than if she just sees me at her office. In those meetings, we talk about almost everything. It is of great importance that the GP participate. Otherwise, she would have had no insight. I am not that often at the GP's office.

Some GPs emphasized that collaborating with health visitors in preventive child health networks provided them with knowledge about local societies and the families' daily lives. House calls could also give important information and generally made it easier to become aware of the children's situation. Most importantly, however, continuity of care gave the GP gradual insights into the families and their social situations. Some GPs did not participate in multidisciplinary meetings, but those who did experienced that they could also support the parents this way.

#### Building Competence

In the survey (paper 4), the participant GPs were asked about what would help them to ensure the support of these vulnerable families. In particular, younger GPs and GPs who were not specialists in general practice reported “more competence about children as next of kin.” Some of the GPs wanted more training in talking to children about sensitive matters. Notably, many of them did not know about the 2010 law (15) that requires them to, given the parents' informed consent, ensure that children receive information and follow-up. The GPs called for, among other things, net-based courses, booklets, and overviews of the services for families in primary care and social services.

## Discussion

The parents and the GPs who participated in the four sub-studies generally accepted that the parents' health problems might have a negative impact on their children's current and future wellbeing, health, and psychosocial adaptation. Despite this, both groups were ambivalent about addressing the children's situations during encounters in doctors' offices, and the topic was often omitted. This analysis suggests recommendations for how GPs can overcome these barriers: namely (1) recognize the parents' good intentions, (2) ask directly about their children, (3) learn more about children as next of kin, (4) learn more about how to talk to children, (5) build a trusting alliance with the parents and rely on it, and (6) participate in multidisciplinary networks concerning these families. Some of these elements are previously described from general practice and specialist health services (10, 29, 30, 42). Our research, however, points out opportunities to incorporate these elements into a clinical context in general practice.

Many authors recommend using a family-focused approach in general practice to reach children in need (26, 29). Based on a qualitative study of GPs in Denmark, Holge-Hazelton and Tulinius (19) defined “cases with a child in need” in general practice as “a case that directly or indirectly involves problems with a specific child, an as-yet unborn child, or one or both parents of a family, currently or potentially threatening the wellbeing of the family and the child.” They found that in general practice, most cases with a child in need are found during indirect consultations; indirect in the sense that the child is not present, or the primary cause of the consultation itself may not be the child. This is in accordance with the aim of our study: to indirectly support children as next of kin during consultations with their parents. However, the results from the present analyses suggest that a family focus is a necessary, but not sufficient prerequisite. In addition, the GP usually has to prompt parents to bring up the children's situations and overcome the ambivalent feelings both in themselves and the parents.

Our results showed that the GPs were often unsure whether the parent tolerated their inquiry into family matters. Their concern was that some parents might think this topic was not a GP's business, be offended, leave the office and—in the worst case—leave their patient registry. Broholm-Jorgensen et al. (43) recently published a study on GPs' strategies for retaining patients during preventive health checks. Opportunistic health checks during consultations for other topics might be provocative for patients. The researchers found that respect was a core element for the GP to succeed in a professional urge to promote better health practices in smokers, overweight persons, etc. They identified two complementary fields of respect: the GP's respect for the patient's autonomy and the patient's respect for the GP's professional authority. If the GP balanced the emphasis on his or her authority with their respect for the patient's autonomy, there was an increased chance that the patient would come back for another consultation. During such consultations, there would ideally be an exchange of mutual respect. Because GPs have ethical and legal obligations to address children of parents with illnesses and substance abuse during consultations with these parents, it is crucial—given the result from the aforementioned study—that GPs treat the topic of children with respect. Trust and recognition might be useful concepts to apply here as well.

The parents wanted information and advice from a trusted helper from whom they could accept direct speech and alternative viewpoints. This lead into the question of how the GP can evaluate the strength and quality of the patient's trust. Skirbekk et al. (36), having studied GPs' consultations, concluded that doctor-patient trust is mostly indirectly, and rarely openly, addressed by doctor and patient. The patient gives the doctor a “mandate of trust” in which the patient sets the conditions for what is an accepted topic during the consultation. This mandate can be limited or broad, and it may change during the consultation. The GP might negotiate the mandate of trust by taking the initiative to talk openly about the patient–doctor relationship. To what extent does the patient trust the GP? What are his or her doubts and mistrust if the topic of the children's situation is addressed? An open talk might disclose that the patient already indirectly has given the GP an open mandate, or that the patient does not have enough trust in the doctor to reveal problems at home that might affect the children. Then these concerns might become a topic in the consultation.

As the study of Broholm-Jorgensen et al. demonstrated (43), recognition may be a crucial aspect of the exchange of mutual respect. When asked about daily family lives, most parents in the present study first emphasized how well they managed their ordinary routines. Later, some revealed difficulties, such as worries about their children and needing support for themselves and their children. As described in the section “Theoretical assumptions,” an attitude of recognition allows for the acceptance of another person's experiences and opinions. This attitude has been shown to be beneficial to a doctor–patient relationship in which the patient may gradually accept the doctor's viewpoints. For example, after the parent has told the GP about the efforts that he or she puts into maintaining as ordinary a life as possible for the children and the GP has verbally recognized this efforts, the parent may respond and give the GP trust in return and respect the GPs authority and advice about the children's need for support. However, the GP is responsible for laying the groundwork for this process to develop.

If the GPs manage to overcome the mutual ambivalence to address the family situation during consultations, this might benefit not only the children but also the parents with impairments. In a Dutch interview study of parents with mental health and substance abuse disorders (44), the parents stated that parenthood and the demands of parenting gave meaning and structure to their lives, and thereby provided them with strength. Therefore, if the GPs come in a position to support and coach parents by taking the parents' experiences and goals as a starting point, this will be of help for both children and parents. In the parental interviews (Paper 3), we learned how important their identity as responsible parents was, but also their need for emotional and practical support.

The GPs wanted to build more competence in the topic of children as next of kin, and stated that this could raise their awareness and ensure adequate support for the children. However, the topic is inconsistently referred to in the medical education curriculum and continuing education for specialists in general practice. Therefore, medical schools, governmental health authorities, and the Norwegian Medical Association must take measures to prevent transgenerational transference of psychosocial problems. For example, child-focused and family-focused content should be strengthened in the training of medical students as well as in the continuing education for GPs, including ways to overcome barriers in raising potentially confrontational issues about children's welfare during consultations.

### Strengths and Limitations

To our knowledge, the research project Burdened Children as Next of Kin and the General Practitioner is one of very few focusing on the care of these children in general practice. Because GPs are central to the primary health care of the parents in the target groups and their children, our project may act as a basis for clinical work and further research. It is a strength of the project that it combines experiences from all three relevant informant groups: children, parents, and GPs.

The empirical material for this analysis was a text composed of the result sections of the four published articles and not the original raw data from the four projects. This may constitute a limitation regarding richness. However, the aim of the present secondary analysis was to gain further knowledge about the consultation situation, where the main experiences of the actors were juxtaposed and abstracted into salient themes. This bird's eye view of the situation was possible only by looking at the main experiences after they had been identified in the previous sub-studies. That said, there were some limitations in the data collection methods used in the sub-studies. First, the parents were recruited through their GPs, and because of this it is possible that they had more positive experiences with their doctor than informants on average. We have little information from those who could reveal shortcomings in GPs' services, did not have a rapport with their GP, or had their health services covered by specialist services. Second, the material contains no direct information from “invisible” children because the adolescents were recruited from support groups. Some of them, however, told stories in retrospect about their daily lives before they were identified and helped. Third, the results section of paper 4 refers to 499 respondents to a survey sent to ~6,000 GPs. We can assume that the results then are based on answers from GPs who were more interested in the topic that the average GP (45).

Finally, some interviewer and interpreter biases may have occurred because the interviewers were GPs (FG) and child and adolescent psychiatrists (MH). How the participants presented themselves and their experiences might have been influenced by this fact (46). The GPs may have avoided telling stories about encounters where they felt that they failed and the adolescents and the parents generally avoided sharing bad experiences from encounters with GPs.

Overall, the material possibly contains less information about bad experiences of encounters with GPs where the children's life situations were addressed. We assume that there are more aspects concerning these encounters than our analysis has brought forward. Nevertheless, the suggestions we have provided for GPs to overcome mutual ambivalences during these encounters are valid and can be transferred to general practices in Norway and most likely to comparable GP and health care systems.

### Implications and Advice for the GP (Memory box)

- Assume that parents are striving for and want the best for their children. Acknowledge parents' efforts to maintain ordinary daily lives for their children.
- Keep in mind that most of these parents want their children's situations to be addressed, but the parents must be prompted by a GP that they trust.
- Conduct critical evaluation of the doctor–patient relationships with parents. Undertake necessary efforts to obtain a working mandate of trust that ensures openness about the children's situations.
- Expand personal knowledge and skills on the topic, including the local psychosocial networks of families with problems, relevant support resources, and everyday challenges children as next of kin are facing.
- Give priority to and actively participate into collaborations with the other helpers for a specific family.
- Establish contact with support services. Provide children and parents with a list of possible support services for families with children as next of kin.

## Further Research

It is important to continue the work to develop evidence-based guidelines for GPs during encounters with parents with illnesses and/or substance abuse to ensure their children adequate information about their parent's impairments and follow-up if necessary. The following specific recommendations for research are:

1. A web-based survey with a representative sample of Norwegian GPs. The aim of the survey would be to assess (1) the distribution of views and attitudes and current practices related to what most Norwegian GPs consider to be good service to burdened children and their families in general practice, (2) how the expectations of the children and their parents (Paper 2 and 3) can be met, and (3) GPs evaluation of how the preliminary guidelines from the present sub-study can be applied in their practices.
2. A multicenter randomized controlled trial with GPs in Scandinavia. The intervention could be education on the topic of children as next of kin and clinical training in applying the preliminary guidelines from the present sub-study. The outcome measure would be if children of the GPs' patients are identified and offered follow-up. Are there differences between the intervention group and the control group? We plan to include centers in Sweden and Denmark to achieve a sample that is large enough to give statistically significant results. The context for general practice in Scandinavia is fairly equal.

## Ethical Approval

This study was carried out in accordance with recommendations of the Western Norway Regional Authority and theResearch Council of Norway. The empirical material in the present article stem from our previous four articles in the project. Two of the articles (10, 30) received approval from Regional Committee for Medical and Health Research Ethics, Western Norway (2012/2336-3). According to the Regional Committee for Medical and Health Research Ethics, the Act does not apply to the sub-studies published in the two other articles (31, 32) and the present article. All subjects gave a written informed consent in accordance with the Declaration of Helsinki.

## Availability of Data and Material

All audiotapes and transcripts are stored in secure, password-protected storage at the University of Bergen. De-identified transcripts from the interviews may be made available to interested persons or organizations on request to the corresponding author at Marit.Hafting@uni.no.

## Author Contributions

MH headed the research group Children as Next of Kin and the General Practitioner and had formal responsibility in all stages of the present research process. FG and MH coded the material in the present article. FG and KvD followed the research process closely and commented during the work process. MH, FG, NA, TS-S, and GR worked together on the foundation of the project and had substantial contributions to the four published articles (10, 30–32). NA, TS-S, and GR commented on the last versions of the present article, and all the authors approved it.

### Conflict of Interest Statement

The authors declare that the research was conducted in the absence of any commercial or financial relationships that could be construed as a potential conflict of interest.
